# Significant impact of body mass index on the relationship between increased white blood cell count and new-onset diabetes

**DOI:** 10.7150/ijms.80207

**Published:** 2023-01-31

**Authors:** Chieh-Yu Hsieh, Wen-Hsien Lee, Yi-Hsueh Liu, Chun-Chi Lu, Szu-Chia Chen, Ho-Ming Su

**Affiliations:** 1 Department of Internal Medicine, Kaohsiung Municipal Siaogang Hospital, Kaohsiung Medical University Hospital, Kaohsiung Medical University, Kaohsiung, Taiwan.; 2 Division of Hematology and Oncology, Department of Internal Medicine, Kaohsiung Medical University Hospital, Kaohsiung Medical University, Kaohsiung, Taiwan.; 3 Division of Cardiology, Department of Internal Medicine, Kaohsiung Medical University Hospital, Kaohsiung Medical University, Kaohsiung, Taiwan.; 4 Division of Nephrology, Department of Internal Medicine, Kaohsiung Medical University Hospital, Kaohsiung Medical University, Kaohsiung, Taiwan.; 5 Faculty of Medicine, College of Medicine, Kaohsiung Medical University, Kaohsiung, Taiwan.

**Keywords:** white blood cell count, body mass index, new-onset diabetes

## Abstract

An elevated white blood cell (WBC) count has been linked to incident diabetes. WBC count has been positively associated with body mass index (BMI), and elevated BMI has been reported to be a strong predictor of future diabetes. Hence, the association of increased WBC count with the subsequent development of diabetes may be mediated by increased BMI. This study was designed to address this issue. We selected subjects from the 104,451 participants enrolled from 2012 to 2018 in the Taiwan Biobank. We only included those with complete data at baseline and follow-up and those without diabetes at baseline. Finally, 24,514 participants were enrolled in this study. During an average 3.88 years of follow-up, 248 (1.0%) of the participants had new-onset diabetes. After adjusting for demographic, clinical, and biochemical parameters, increased WBC count was associated with new-onset diabetes in all of these participants (*p* ≤ 0.024). After further adjustment for BMI, the association became insignificant (*p* = 0.096). In addition, subgroup analysis of 23,430 subjects with a normal WBC count (range: 3500-10500/µl) demonstrated that increased WBC count was significantly associated with new-onset diabetes after adjusting for demographic, clinical, and biochemical parameters (*p* ≤ 0.016). After further adjustment for BMI, this association was attenuated (*p* = 0.050). In conclusion, our results showed that BMI had a significant impact on the relationship between increased WBC count and new-onset diabetes in all study participants, and BMI also attenuated the association in those with a normal WBC count. Hence, the association between increased WBC count and the future development of diabetes may be mediated by BMI.

## Introduction

Chronic inflammation has been demonstrated to play a key role in the pathogenesis of type 2 diabetes mellitus (DM) 1. Inflammation itself has been shown to cause insulin resistance 2 and promote β-cell death 3. Previous studies have suggested an association between white blood cell (WBC) count, a non-specific parameter of inflammation, and incident DM 4,5. However, in contrast to these findings, Chao et al. found that measurements of plasma markers of systemic inflammation, including WBC count, contributed little additional value in predicting the risk of DM 6. In addition, several studies have demonstrated a significant link between C-reactive protein (CRP), a useful marker of inflammation, and incident DM after adjusting for obesity indexes 7,8, whereas other studies have argued that such associations may be chiefly mediated by increased adiposity 6. Thorand et al. reported that the association between an increased CRP level with future DM risk became insignificant after adjusting for body mass index (BMI) 9. Hence, BMI may have a significant influence on the relationship between chronic inflammation and future development of DM.

WBC count has been positively associated with BMI 10,11, and increased BMI has been shown to be a strong predictor of future DM in various populations, including 29,136 Swedish twins 12, 211,833 Chinese adults >20 years old 13, and 88,305 Japanese subjects 14. Hence, the association of increased WBC count with the subsequent development of DM may be mediated by increased BMI. This study was designed to evaluate whether BMI has a significant impact on the relationship between WBC count and new-onset DM.

## Methods

### Study Population

Our study subjects were collected from the Taiwan Biobank (TWB), a general population-based research database including cancer-free residents aged 30-70 years selected from 31 recruitment stations in Taiwan since 2008. Details of the TWB have been described previously 15,16. The methodologies of data collection from all participants in the TWB were identical and followed a standardized process. Details about the TWB can be found on its official website (https://taiwanview.twbiobank.org.tw/index). Written informed consent was obtained from all enrolled participants, and this study was conducted according to the Declaration of Helsinki. This study was approved by the Institutional Review Board of Kaohsiung Medical University Hospital (KMUHIRB-E(I)-20180242) on March 8, 2018.

We selected study subjects from the 104,451 participants enrolled from 2012 to 2018 in the TWB. Demographic data including age, sex, smoking history, and history of DM and hypertension, were obtained from a face-to-face interview with TWB investigators. BMI, systolic blood pressure, resting heart rate, and overnight fasting blood chemistry parameters, including fasting blood glucose, total cholesterol, triglycerides, uric acid, serum creatinine, hematocrit, WBC count, platelet count, and hemoglobin A1c (HbA1c) were collected. All of these data were acquired at baseline and at a mean ± standard deviation follow-up period of 3.88 ± 1.16 years. The enrolled participants were followed up after 2-4 years. Information including the results of a questionnaire, physical examination and blood examination were collected upon enrollment and at the follow-up visit. We only selected participants with complete data at baseline and follow-up (n = 27,209). Patients with DM at baseline (n= 2695) were excluded. Finally, 24,514 participants without DM at baseline (8392 men and 16,122 women) were included in this study.

### Definition of subjects without diabetes

Subjects who had not received anti-diabetic medications, had no past history of DM, and whose fasting blood glucose was less than 126 mg/dl and HbA1c was less than 6.5% were considered not to have DM.

### Statistical analysis

SPSS 22.0 for Windows (SPSS Inc. Chicago, USA) is used to perform statistical analysis. Data are expressed as mean (standard deviation) or number (percentage), as applicable. Differences in continuous and categorical variables between groups are compared using the independent samples t-test and chi-square test, respectively. Normality tests are done to analyze the distribution of data collected for each group using the Kolmogorov-Smirnov test. Homogeneity of variance is tested with Levene's test (Levene's test is used to assess the equality of variance along with an independent sample t-test). Univariate binary logistic analysis is used to identify the factors associated with new-onset DM. Statistically significant variables in univariate binary logistic analysis are selected into multivariate binary logistic analysis, which is performed using a modified stepwise procedure in four modeling steps. The first model consists of age and sex. The second model adds significant clinical risk factors in the univariate analysis (hypertension, smoking history, systolic blood pressure, and heart rate). The third step adds significant laboratory data in the univariate analysis (fasting blood glucose, HbA1c, eGFR, uric acid, triglycerides, and hematocrit). The final step adds BMI to the model. Multivariate linear regression analysis is used to identify the major determinants of baseline WBC count. The results of binary logistic analysis are expressed as odds ratio (OR) and 95% confidence interval (CI). The results of linear regression analysis are expressed as standardized coefficient β. Receiver operating characteristic (ROC) curve analysis and areas under the ROC curves (AUCs) are used to assess the performance and predictive ability of WBC count for new-onset DM. Optimal cutoff values are those with the highest Youden index, or equivalently, the highest sensitivity + specificity. The POWER procedure using SAS statistical software (version 9.4, SAS Institute, Cary, NC, USA) performs power and sample size analyses. Mediation analysis is performed using the CAUSALMED procedure, new in SAS/STAT 14.3. A two-tailed *p* value less than 0.05 was considered statistically significant.

## Results

**Table 1** shows comparisons of baseline characteristics between the participants with and without new-onset DM in all 24,514 subjects. Compared to those without new-onset DM, those with new-onset DM were older, more predominantly male, had a higher prevalence of hypertension history, higher prevalence of smoking history, higher systolic blood pressure and heart rate, higher fasting blood glucose, HbA1c, uric acid, triglycerides, WBC count, hematocrit, and BMI, and lower estimated glomerular filtration rate (eGFR) at baseline.

**Table 2** shows the ORs of variables associated with new-onset DM in univariate binary logistic analysis in all study participants. Older age, male sex, hypertension history, smoking history, increased systolic blood pressure, heart rate, fasting blood glucose, HbA1c, uric acid, triglycerides, WBC count, hematocrit and BMI, and decreased eGFR were associated with new-onset DM.

**Table 3** shows the ORs for the association of WBC count with new-onset DM in multivariate binary logistic analysis in all study participants. The mean follow-up period was 3.88 ± 1.16 years in all patients, during which 248 (1.0%) subjects developed DM. Increased WBC count was significantly associated with new-onset DM in the age- and sex-adjusted model (*p* = 0.001) and in the multivariate model adjusting for age, sex, hypertension, smoking history, systolic blood pressure, and heart rate (*p* = 0.002). This relationship remained significant after further adjustments for significant laboratory data in the univariate analysis, including fasting blood glucose, HbA1c, eGFR, uric acid, triglycerides, and hematocrit (*p* = 0.024). However, the relationship became insignificant after further adjustment for BMI (*p* = 0.096).

We have performed post hoc power analysis. This investigation at the predictor variable (WBC) had a statistical power of 84% to detect an OR of 1.118 at α=0.10. We have further performed mediation analysis. The 'Percentage Mediated' change is 4.21% (*p* = 0.0775). When the interaction term is included, the 'Percentage Mediated' changes slightly from 4.21% (*p* = 0.0775) for the model without this term) to 4.48% (*p* = 0.0675). The percentage due to interaction is not significant (*p* = 0.362).

**Table 4** shows the ORs for the association of WBC count with new-onset DM in multivariate binary logistic analysis in different subgroups of participants. In the subgroup of 23,430 participants with a normal WBC count (range, 3500-10500/µl^17^), increased WBC count was significantly associated with new-onset DM in the unadjusted model (*p* < 0.001), in the age- and sex-adjusted model (*p* < 0.001), and in the multivariate model adjusting for age, sex, hypertension, smoking history, systolic blood pressure, and heart rate (*p* < 0.001). This relationship remained significant after further adjustments for significant laboratory data in the univariate analysis, including fasting blood glucose, HbA1c, eGFR, uric acid, triglycerides, and hematocrit (*p* = 0.016). However, the relationship was attenuated after further adjustment for BMI (*p* = 0.050). In the subgroup of participants with BMI > 25 kg/m^2^ (n = 8064), increased WBC count was significantly associated with new-onset DM in the unadjusted model (*p* = 0.009), in the age- and sex-adjusted model (*p* = 0.001), and in the multivariate model adjusting for age, sex, hypertension, smoking history, systolic blood pressure, and heart rate (*p* = 0.009). However, the relationship became borderline significant after further adjustments for significant laboratory data in the univariate analysis, including fasting blood glucose, HbA1c, eGFR, uric acid, triglycerides, and hematocrit (*p* = 0.059). Moreover, the relationship became insignificant after further adjustment for BMI (*p* = 0.112). In the subgroup of participants with BMI ≤ 25 kg/m^2^ (n = 16,450), increased WBC count was not associated with new-onset DM in the unadjusted model (*p* = 0.246).

**Table 5** shows the standardized coefficients of variables associated with WBC count in univariate and multivariate linear regression analyses in all study participants. Results of the multivariate analysis showed that increased WBC count was significantly associated with younger age, female sex, presence of hypertension, smoking history, increased systolic blood pressure, heart rate, HbA1c, uric acid, triglycerides, hematocrit, platelet count and BMI, and decreased total cholesterol.

The performance (ROC curves) and predictive ability (AUCs) of WBC count to identify new-onset DM were analyzed. The AUC of WBC count was 0.584 (95% CI: 0.548-0.619, *p* < 0.001). The cutoff value of WBC count was 5950/µl, and the sensitivity and specificity of this cutoff value were 56.0% and 55.9%, respectively.

## Discussion

In this study, we evaluated the association of WBC count with new-onset DM in 24,514 non-diabetic subjects during a mean 3.88 years of follow-up. We found that increased WBC count was significantly associated with new-onset DM in unadjusted and several multivariate models, but that this association became insignificant after further adjustment for BMI in all study participants. BMI had a great impact on the relationship between increased WBC count and new-onset DM. In addition, we performed subgroup analyses, and found that in the participants with normal WBC count, increased WBC count could predict new-onset DM in the unadjusted and all multivariate models. Further adjustment for BMI did not alter the association between increased WBC count and new-onset DM. In addition, in the participants with BMI > 25 kg/m^2^, WBC count was positively associated with new-onset DM in the unadjusted and several multivariate models, but this association became insignificant after further adjustment for BMI. Hence, BMI had also a significant impact on the relationship between increased WBC count and new-onset DM in the participants with BMI > 25 kg/m^2^. Finally, in the participants with BMI ≤ 25 kg/m^2^, WBC count could not predict new-onset DM, even in the unadjusted model.

The results of previous studies have been inconsistent with regards to whether increased WBC count contributes to DM prediction models independently of obesity 4,5 or whether elevated WBC count only reflects an increase in adipose tissue mass 6. Increased BMI has been shown to be an essential contributor to DM through insulin resistance and islet β-cell failure 2,3,18,19. Kashima et al. reported that increased WBC count was predictive of type 2 DM, and that the combination of increased WBC count and BMI augmented the risk of DM, regardless of whether BMI was high or low 4. In addition, Gu et al. reported that WBC count could be used as an indicator to identify whether or not obesity led to an increased risk of DM 20. In contrast, Oda demonstrated that WBC count could not independently predict incident DM in a Japanese health screening population in whom obesity was not prevalent 21. Chao et al. evaluated the utility of inflammation markers to predict the risk of type 2 DM in 93,676 women aged 50 to 79 years, and found that beyond traditional risk factors, WBC count contributed relatively little additional predictive value 6. Hence, whether or not WBC count is a useful predictor of incident DM is unclear. Twig et al. assessed whether WBC count was an independent risk factor for DM among 185,354 young healthy adults using a multivariate model adjusted for age, BMI, family history of diabetes, physical activity, and fasting glucose and triglycerides levels, and revealed a 7.6% increase in incident DM for every 1000/µl increase in WBC count They further found that after controlling for risk factors, BMI was the primary contributor to the variation in the multivariate models for the prediction of incident DM 22. This finding is similar to ours, in that the significant relationship between increased WBC count and new-onset DM became insignificant after adjusting for BMI in all study participants and in the subgroup of participants with BMI > 25 kg/m^2^. In addition, Twig et al. also found that WBC count was not associated with an increased risk of DM in lean and normoglycemic men with BMI < 25 kg/m^2^ 22, which is also consistent with our subgroup analysis, i.e. no association between WBC count and new-onset DM in the subjects with BMI ≤ 25 kg/m^2^. We postulate that the pathogenesis may be characterized by an increased BMI leading to inflammation, as expressed by an increased WBC, resulting in impaired glucose tolerance. However, mediation analysis reveals that the percentage due to interaction is not significant, which means that the interpretation of the results is not significant due to the mediation of WBC and is not drastically different from those of the analysis with no interaction. Further studies are still needed to elucidate the causal relationship, and clarify whether WBC is an independent risk factor for the development of DM, as it is difficult to determine whether WBC is a pathogenic mediator or a marker.

Leukocytosis, a common laboratory finding, can be caused by infections and inflammatory processes 23, physical and emotional stress 24,25, and some medications such as corticosteroids, lithium, and beta agonists 26-28. Leukopenia is probably caused by certain medications 29, autoimmune diseases 30 and neoplasia 30. We performed a subgroup analysis of 23,430 subjects with normal WBC count (range, 3500-10500/µl) and found that although the relationship was also attenuated after adjusting for BMI, increased WBC count was still a useful predictor of the future development of DM in the multivariate analysis. Hence, after excluding subjects with abnormally low and high WBC counts, our results demonstrated that increased WBC count was useful in predicting the future development of DM.

Previous studies have reported a positive association between WBC count and metabolic syndrome as well as hypertriglyceridemia, low HDL-cholesterol, high fasting glucose, and all components of the metabolic syndrome 31-33. In this study, we similarly demonstrated that increased WBC count was significantly associated with the presence of hypertension and increased systolic blood pressure, HbA1c, triglycerides, and BMI. Chen et al. examined the association between WBC count and risk of coronary heart disease in middle-aged and elderly patients with hyperuricemia, and found a significant reverse correlation between tertiles of WBC count and age 34. In the present study, we also found that WBC count was negatively correlated with age in the multivariate analysis. An elevated resting heart rate has also been associated with an increased WBC count 35,36, which is consistent with our findings. In addition, Liu et al. found that WBC count was positively associated with uric acid, and that this association was independent of conventional risk factors 37. Our results also showed that WBC count was positively correlated with uric acid.

There were several limitations to this study. First, our study participants were included from the TWB, which does not include information on medications. The use of anti-hypertensive medications, lipid lowering agents, and hypouricemic agents would have influenced the values of blood pressure, resting heart rate, lipid profile, and uric acid. Therefore, we could not exclude the impact of such medications on our results. Second, we also lacked data on differential WBC count (lymphocytes, monocytes, neutrophils, etc.), and clusters of differentiation (CD) (CD4, CD8, CD14, CD16, CD20, CD45, etc.), so we could not analyze the relationship between differential count of WBC and CD with new-onset DM. In addition, although we used inflammation to link WBC and DM, we lacked CRP data to validate the relationship. Further longitudinal studies are warranted to investigate the relationships among differential WBC count, CD, and CRP with new-onset DM. Finally, high WBC count may reflect acute infection, tissue damage, and other inflammatory conditions, and low WBC count be caused by other comorbidities or malnutrition, which were not recorded in our data set.

In conclusion, our results showed that BMI had a significant impact on the relationship between increased WBC count and new-onset DM. In all study subjects and in the obese group, BMI had a significant impact on association between increased WBC count and new-onset DM, and BMI also attenuated the association in those with a normal WBC count. Hence, the association between increased WBC count and the future development of diabetes may be mediated by BMI.

## Figures and Tables

**Table 1:** Comparison of baseline characteristics between participants with and without new-onset DM

Characteristics	Participants with new-onset DM (n = 248)	Participants without new-onset DM (n= 24266)	*p* value	All participants (n = 24514)
Age (year)	55 ± 9	51 ± 10	<0.001	51 ± 10
Male (%)	44.8	34.1	<0.001	34.2
Hypertension (%)	29.4	10.7	<0.001	10.9
Smoking history (%)	23.5	29.8	0.019	23.6
Systolic blood pressure (mmHg)	125 ± 17	116 ± 17	<0.001	117 ± 17
Heart rate (beat/min)	71 ± 11	69 ± 9	<0.001	69 ± 9
Fasting blood glucose (g/dl)	103 ± 10	92 ± 8	<0.001	92 ± 8
HbA1c (%)	6.00 ± 0.32	5.57 ± 0.34	<0.001	5.57 ± 0.34
eGFR (ml/min/1.73m^2^)	105 ± 26	110 ± 25	0.005	110 ± 25
Uric acid (mg/dl)	6.0 ± 1.5	5.4 ± 1.4	<0.001	5.4 ± 1.4
Total cholesterol (mg/dl)	197 ± 38	196 ± 35	0.761	196 ± 35
Triglyceride (mg/dl)	160 ± 134	109 ± 76	<0.001	110 ± 77
WBC (1000/µl)	6.35 ± 1.61	5.92 ± 1.60	<0.001	5.92 ± 1.60
Hematocrit (%)	43.9 ± 4.5	43.0 ± 4.5	0.004	43.1 ± 4.5
Platelet (1000/µl)	240 ± 80	242 ± 57	0.614	242 ± 58
BMI (kg/m^2^)	26.2 ± 3.9	23.8 ± 3.4	<0.001	23.9 ± 3.4

BMI: body mass index; DM, diabetes mellitus; eGFR, estimated glomerular filtration rate; HbA1c, hemoglobin A1c; WBC, white blood cell.

**Table 2:** Odds ratio (95% CI) of variables associated with new-onset diabetes in univariate binary logistic analysis in all study participants (n = 24514)

Parameter	OR (95% CI)	*P*
Age (per 1 year)	1.049 (1.035-1.063)	<0.001
Male (*vs.* female)	1.564 (1.216-2.012)	<0.001
Hypertension	3.473 (2.635-4.577)	<0.001
Smoking history	1.384 (1.053-1.820)	0.020
Systolic blood pressure (per 1 mmHg)	1.024 (1.017-1.030)	<0.001
Heart rate (per 1 beat/min)	1.022 (1.010-1.034)	<0.001
Fasting blood glucose (per 1 g/dl)	1.144 (1.129-1.158)	<0.001
HbA1c (per 0.1%)	1.517 (1.452-1.585)	<0.001
eGFR (per 1 ml/min/1.73m^2^)	0.992 (0.987-0.998)	0.004
Uric acid (per 1 mg/dl)	1.281 (1.183-1.386)	<0.001
Total cholesterol (per 1 mg/dl)	1.001 (0.997-1.004)	0.761
Triglyceride (per 1 mg/dl)	1.003 (1.002-1.004)	<0.001
WBC (per 1000/µl)	1.107 (1.048-1.170)	<0.001
Hematocrit (per 1%)	1.043 (1.014-1.072)	0.004
Platelet (per 1000/µl)	0.999 (0.997-1.002)	0.614
BMI (per 1 kg/m^2^)	1.163 (1.131-1.196)	<0.001

CI, confidence interval; OR, odds ratio; other abbreviations as in Table 1.

**Table 3:** Odds ratio (95% CI) of WBC count (per 1000/µl) with new-onset diabetes in multivariate binary logistic analysis in all study participants (n = 24514)

	OR (95% CI)	*p*
Unadjusted	1.107 (1.048-1.170)	<0.001
Age and sex adjusted	1.118 (1.045-1.197)	0.001
Multivariate adjusted (1)	1.087 (1.030-1.147)	0.002
Multivariate adjusted (2)	1.082 (1.010-1.158)	0.024
Multivariate adjusted (3)	1.066 (0.989-1.150)	0.096

CI, confidence interval; OR, odds ratio; other abbreviations as in Table 1. Multivariate model (1): adjusted for age, sex, hypertension, smoking history, systolic blood pressure, and heart rate. Multivariate model (2): model (1) + significant laboratory data in the univariate analysis, including fasting blood glucose, HbA1c, eGFR, uric acid, triglyceride, and hematocrit. Multivariate model (3): model (2) + BMI.

**Table 4:** Odds ratio (95% CI) of WBC count (per 1000/µl) with new-onset diabetes in multivariate binary logistic analysis in subgroup participants

Subgroups	OR (95% CI)	*p*
**Subgroup A: participants with 3500 < WBC count < 10500/µl (n = 23430)**		
Unadjusted	1.208 (1.108-1.317)	<0.001
Age and sex adjusted	1.243 (1.138-1.358)	<0.001
Multivariate adjusted (1)	1.182 (1.080-1.295)	<0.001
Multivariate adjusted (2)	1.129 (1.022-1.246)	0.016
Multivariate adjusted (3)	1.106 (1.000-1.223)	0.050
**Subgroup B: participants with BMI > 25 kg/m^2^ (n = 8064)**		
Unadjusted	1.131 (1.032-1.241)	<0.001
Age and sex adjusted	1.173 (1.069-1.287)	0.001
Multivariate adjusted (1)	1.135 (1.032-1.249)	0.009
Multivariate adjusted (2)	1.105 (0.996-1.227)	0.059
Multivariate adjusted (3)	1.090 (0.980-1.213)	0.112
**Subgroup C: participants with BMI ≤ 25 kg/m^2^ (n = 16450)**		
Unadjusted	1.052 (0.966-1.146)	0.246

CI, confidence interval; OR, odds ratio; other abbreviations as in Table 1. Multivariate model (1): adjusted for age, sex, hypertension, smoking history, systolic blood pressure, and heart rate. Multivariate model (2): model (1) + significant laboratory data in the univariate analysis, including fasting blood glucose, HbA1c, eGFR, uric acid, triglyceride, and hematocrit. Multivariate model (3): model (2) + BMI.

**Table 5:** Standardized coefficient of variables associated with WBC count in univariate and multivariate linear regression analyses in all study participants (n = 24514)

Parameters	WBC count			
	Univariable analysis		Multivariable analysis	
	β	*p*	β	*P*
Age (per 1 year)	-0.134	0.001	-0.103	< 0.001
Male (*vs.* female)	0.126	<0.001	-0.028	0.001
Hypertension (%)	0.057	<0.001	0.028	< 0.001
smoking history	0.138	< 0.001	0.070	< 0.001
Systolic blood pressure (per 1 mmHg)	0.086	<0.001	0.037	< 0.001
Heart rate (per 1 beat/min)	0.145	<0.001	0.093	< 0.001
Fasting blood glucose (per 1 g/dl)	0.007	0.303	-	-
HbA1c (per 0.1%)	0.061	<0.001	0.034	< 0.001
eGFR (per 1 ml/min/1.73m^2^)	-0.031	<0.001	-0.013	0.055
Uric acid (per 1 mg/dl)	0.175	<0.001	0.036	< 0.001
Total cholesterol (per 1 mg/dl)	0.017	0.009	-0.033	< 0.001
Triglyceride (per 1 mg/dl)	0.166	<0.001	0.057	< 0.001
Hematocrit (per 1%)	0.183	<0.001	0.181	< 0.001
Platelet (per 1000/µl)	0.277	<0.001	0.284	< 0.001
Body mass index (per 1 kg/m^2^)	0.235	<0.001	0.115	< 0.001

β: standardized coefficient; other abbreviations as in Table 1.

## References

[1] Poznyak A, Grechko AV, Poggio P, Myasoedova VA, Alfieri V, Orekhov AN (2020). The Diabetes Mellitus-Atherosclerosis Connection: The Role of Lipid and Glucose Metabolism and Chronic Inflammation. International journal of molecular sciences.

[2] Zand H, Morshedzadeh N, Naghashian F (2017). Signaling pathways linking inflammation to insulin resistance. Diabetes & metabolic syndrome.

[3] Usmani-Brown S, Perdigoto AL, Lavoie N, Clark P, Korah M, Rui J (2019). β cell responses to inflammation. Molecular metabolism.

[4] Kashima S, Inoue K, Matsumoto M, Akimoto K (2019). White Blood Cell Count and C-Reactive Protein Independently Predicted Incident Diabetes: Yuport Medical Checkup Center Study. Endocrine research.

[5] Johnston CD, Hoover DR, Shi Q, Sharma A, Hanna DB, Anastos K (2020). White Blood Cell Counts, Lymphocyte Subsets, and Incident Diabetes Mellitus in Women Living With and Without HIV. AIDS research and human retroviruses.

[6] Chao C, Song Y, Cook N, Tseng CH, Manson JE, Eaton C (2010). The lack of utility of circulating biomarkers of inflammation and endothelial dysfunction for type 2 diabetes risk prediction among postmenopausal women: the Women's Health Initiative Observational Study. Archives of internal medicine.

[7] Pradhan AD, Manson JE, Rifai N, Buring JE, Ridker PM (2001). C-reactive protein, interleukin 6, and risk of developing type 2 diabetes mellitus. Jama.

[8] Effoe VS, Correa A, Chen H, Lacy ME, Bertoni AG (2015). High-Sensitivity C-Reactive Protein Is Associated With Incident Type 2 Diabetes Among African Americans: The Jackson Heart Study. Diabetes care.

[9] Thorand B, Löwel H, Schneider A, Kolb H, Meisinger C, Fröhlich M (2003). C-reactive protein as a predictor for incident diabetes mellitus among middle-aged men: results from the MONICA Augsburg cohort study, 1984-1998. Archives of internal medicine.

[10] Kim JA, Park HS (2008). White blood cell count and abdominal fat distribution in female obese adolescents. Metabolism: clinical and experimental.

[11] Desai MY, Dalal D, Santos RD, Carvalho JA, Nasir K, Blumenthal RS (2006). Association of body mass index, metabolic syndrome, and leukocyte count. The American journal of cardiology.

[12] Xu H, Kuja-Halkola R, Chen X, Magnusson PKE, Svensson P, Carrero JJ (2019). Higher body mass index is associated with incident diabetes and chronic kidney disease independent of genetic confounding. Kidney international.

[13] Chen Y, Zhang XP, Yuan J, Cai B, Wang XL, Wu XL (2018). Association of body mass index and age with incident diabetes in Chinese adults: a population-based cohort study. BMJ open.

[14] Okura T, Nakamura R, Fujioka Y, Kawamoto-Kitao S, Ito Y, Matsumoto K (2018). Body mass index ≥23 is a risk factor for insulin resistance and diabetes in Japanese people: A brief report. PloS one.

[15] Fan CT, Lin JC, Lee CH (2008). Taiwan Biobank: a project aiming to aid Taiwan's transition into a biomedical island. Pharmacogenomics.

[16] Lin JC, Fan CT, Liao CC, Chen YS (2018). Taiwan Biobank: making cross-database convergence possible in the Big Data era. GigaScience.

[17] Mitsopoulos E, Lysitska A, Zanos S, Mplatsa A, Alexandrou ME, Kevrekidou S (2020). Normal white blood cell counts predict long-term mortality of hemodialysis patients. International urology and nephrology.

[18] Jiang H, Yan WH, Li CJ, Wang AP, Dou JT, Mu YM (2014). Elevated white blood cell count is associated with higher risk of glucose metabolism disorders in middle-aged and elderly Chinese people. International journal of environmental research and public health.

[19] Sung KC, Ryu S, Sung JW, Kim YB, Won YS, Cho DS (2017). Inflammation in the Prediction of Type 2 Diabetes and Hypertension in Healthy Adults. Archives of medical research.

[20] Gu Y, Hu K, Huang Y, Zhang Q, Liu L, Meng G (2018). White blood cells count as an indicator to identify whether obesity leads to increased risk of type 2 diabetes. Diabetes research and clinical practice.

[21] Oda E (2015). High-sensitivity C-reactive protein, but not white blood cell count, independently predicted incident diabetes in a Japanese health screening population. Acta diabetologica.

[22] Twig G, Afek A, Shamiss A, Derazne E, Tzur D, Gordon B (2013). White blood cells count and incidence of type 2 diabetes in young men. Diabetes care.

[23] Cerny J, Rosmarin AG (2012). Why does my patient have leukocytosis?. Hematol Oncol Clin North Am.

[24] McCarthy DA, Perry JD, Melsom RD, Dale MM (1987). Leucocytosis induced by exercise. Br Med J (Clin Res Ed).

[25] Darko DF, Rose J, Gillin JC, Golshan S, Baird SM (1988). Neutrophilia and lymphopenia in major mood disorders. Psychiatry Res.

[26] Boggs DR, Joyce RA (1983). The hematopoietic effects of lithium. Semin Hematol.

[27] Liles WC, Dale DC, Klebanoff SJ (1995). Glucocorticoids inhibit apoptosis of human neutrophils. Blood.

[28] Shapiro MF, Greenfield S (1987). The complete blood count and leukocyte differential count. An approach to their rational application. Ann Intern Med.

[29] Barreto JN, McCullough KB, Ice LL, Smith JA (2014). Antineoplastic agents and the associated myelosuppressive effects: a review. J Pharm Pract.

[30] Doulatov S, Notta F, Laurenti E, Dick JE (2012). Hematopoiesis: a human perspective. Cell Stem Cell.

[31] Babio N, Ibarrola-Jurado N, Bulló M, Martínez-González M, Wärnberg J, Salaverría I (2013). White blood cell counts as risk markers of developing metabolic syndrome and its components in the PREDIMED study. PloS one.

[32] Pei C, Chang JB, Hsieh CH, Lin JD, Hsu CH, Pei D (2015). Using white blood cell counts to predict metabolic syndrome in the elderly: A combined cross-sectional and longitudinal study. European journal of internal medicine.

[33] Fadini GP, Marcuzzo G, Marescotti MC, de Kreutzenberg SV, Avogaro A (2012). Elevated white blood cell count is associated with prevalence and development of the metabolic syndrome and its components in the general population. Acta diabetologica.

[34] Chen H, Ding X, Li J, Wu Z, Wang Y, He H (2018). White blood cell count: an independent predictor of coronary heart disease risk in middle-aged and elderly population with hyperuricemia. Medicine.

[35] Hasegawa T, Watase H (2003). Association of heart rate with coronary risk factors and increased white blood cell counts in healthy Japanese people. Journal of atherosclerosis and thrombosis.

[36] Inoue T, Iseki K, Iseki C, Kinjo K (2012). Elevated resting heart rate is associated with white blood cell count in middle-aged and elderly individuals without apparent cardiovascular disease. Angiology.

[37] Liu J, Shen P, Ma X, Yu X, Ni L, Hao X (2019). White blood cell count and the incidence of hyperuricemia: insights from a community-based study. Frontiers of medicine.

